# Clinician Perceptions of AI in Care: Cross-Sectional Survey

**DOI:** 10.2196/95296

**Published:** 2026-09-02

**Authors:** Callum Stephenson, Jazmin Eadie, Mohsen Omrani, Nazanin Alavi

**Affiliations:** 1 Department of Psychiatry Faculty of Health Sciences Queen's University Kingston, ON Canada; 2 OPTT Health Toronto, ON Canada; 3 Providence Care Hospital Kingston, ON Canada; 4 Centre for Neuroscience Studies Faculty of Health Sciences Queen's University Kingston, ON Canada; 5 Kingston Health Sciences Centre Kingston, ON Canada

**Keywords:** artificial intelligence, AI, digital health, psychiatry, clinician perspectives, implementation science, ethical AI, telehealth, mental health care, mental health, clinical care

## Abstract

**Background:**

AI is increasingly being explored as a tool to enhance efficiency, access, and diagnostic accuracy in mental health care. However, the perspectives of clinicians, who are central to the delivery and oversight of care, on the use of AI remain underexamined.

**Objective:**

This study aimed to explore clinicians’ perceptions of AI in clinical care, including perceived benefits, risks, barriers to implementation, and training needs.

**Methods:**

A cross-sectional survey was distributed (via email and social media) from August 2024 to November 2024 to mental health professionals in the United States. The survey aimed to capture attitudes toward AI integration, perceived utility, and adoption-related challenges and facilitators in mental health care. Quantitative data were analyzed using descriptive statistics, whereas open-ended responses were analyzed thematically to identify key insights.

**Results:**

Respondents (N=62) were mostly (43/62, 69.4%) not currently using AI in practice. The most frequently endorsed benefits included reduction in time spent in coding and billing (32/60, 53.3%), automated intake (23/61, 37.7%), and improved documentation (23/61, 37.7%) by mental health professionals. However, most expressed discomfort with using AI in patient care, and concerns were raised about inaccurate outputs (47/61, 77%), algorithmic bias (41/61, 67.2%), reduced accessibility (31/61, 50.8%), and job stability (22/61, 36.1%). Barriers to adoption included clinician resistance (46/61, 75.4%), lack of validation (41/61, 67.2%), and challenges with technical integration (26/61, 42.6%). Most respondents (54/61, 88.5%) believed that specialized training in AI ethics and applications was moderately, very, or extremely important for clinicians. Open-ended responses reinforced concerns about dehumanization, cultural insensitivity, ethical accountability, and insufficient technological literacy.

**Conclusions:**

Mental health professionals in this sample viewed AI as a potentially useful adjunct to care but not a replacement. Ethical concerns, limited trust, and a strong emphasis on the human dimensions of therapy suggest that implementation must proceed with caution. Clinician-informed strategies, ethical frameworks, and targeted training are essential to support the responsible and effective integration of AI into mental health practice.

## Introduction

AI is reshaping health care, with applications from diagnostic imaging and predictive analytics to administrative automation and clinical decision support. In mental health care, where demand consistently exceeds supply, AI is viewed as a way to expand access, improve efficiency, and enhance precision [1-5]. AI systems that process large datasets, identify patterns, and generate real-time insights may streamline assessments, support decision-making, and reduce administrative burden [1-5]. Examples include chatbots for low-acuity support, natural language processing for risk detection, and embedded decision support tools in electronic health records [1-5].

The need for scalable solutions is acute amid mental health professional (MHP) shortages, long wait times, and high caseloads, especially in rural and underserved areas [1-5]. Automating routine tasks such as intake, documentation, and symptom tracking could free clinician time for complex, relational care. AI may also enable earlier risk detection, personalized interventions, and more consistent care [1-5], but these opportunities carry significant ethical and practical implications.

Concerns include algorithmic bias from nonrepresentative training data, potentially worsening disparities [6], and “hallucinations” that could undermine diagnosis or treatment [7]. Privacy, informed consent, and interpretability also remain pressing issues, particularly in trust-dependent clinical settings [8]. Given mental health care’s reliance on therapeutic rapport, narrative understanding, and contextualized interpretation [9], MHPs worry that AI could depersonalize care [10] or erode professional roles [10]. Mitigation requires ethical guidelines, transparency, clinical validation, and training; without safeguards, AI risks harming rather than enhancing care.

While research frequently highlights AI’s technical performance and patient outcomes, literature exploring the perspectives of MHPs is still developing. Recent work indicates that, while clinicians in general medical settings may embrace AI for diagnostic and workflow efficiencies, MHPs express unique reservations regarding the therapeutic alliance and relational empathy [11]. MHPs’ acceptance, skepticism, and feedback directly shape implementation success. Their insights reveal specific adoption barriers, including technical limitations, organizational constraints, financial uncertainty, and gaps in AI literacy [12]. Because mental health care relies heavily on narrative understanding rather than objective biomarkers, MHPs are uniquely positioned to identify clinically relevant and ethically problematic applications within their settings. This guidance is essential for developers, health system leaders, and policymakers.

While existing literature has begun to explore psychiatric attitudes toward AI, much of it focuses on specific technologies or lacks representation across different allied mental health professions. This study addresses a critical gap by examining how a diverse sample of licensed US-based MHPs (including psychologists, psychiatrists, social workers, nurse practitioners, and counselors) perceive AI’s role in clinical care. Using a cross-sectional survey with one open-ended item, the research team explored current AI use, readiness for adoption, perceived benefits, ethical concerns, and training needs. By centering MHPs’ perspectives, this study provides an initial exploration into how clinicians navigate the tension between administrative utility and the potential depersonalization of care, offering preliminary insights to guide the development of clinically meaningful ethical strategies.

## Methods

### Ethical Considerations

Ethics approval was obtained from the Queen’s University Health Sciences and Affiliated Teaching Hospitals Research Ethics Board (6041701). Participation was voluntary, anonymous, and uncompensated. Respondents provided informed consent before answering.

### Design

This cross-sectional mixed methods study examined MHPs’ perceptions of AI in clinical care via a structured online survey capturing quantitative and qualitative data. Guided by implementation science and ethical inquiry principles, it explored attitudes toward AI integration, perceived utility, and adoption-related challenges and facilitators.

### Participants and Recruitment

Eligible participants were licensed or trainee MHPs in the United States. Prior AI experience was not required, but participants needed a basic understanding of AI in health care. A mixed nonprobability sampling strategy was used. Email outreach used convenience sampling by distributing the survey through professional networks and mailing lists affiliated with OPTT to reach individuals meeting our specific inclusion criteria. Social media recruitment via LinkedIn used convenience and snowball sampling. Recruitment took place from August 12, 2024, to November 18, 2024. This duration helped maintain stable contextual factors and allowed for multiple recruitment waves without risking major shifts in practice norms. Interested individuals reviewed a letter of information and provided implied consent via survey completion. Email reminders were sent monthly, and social media posts were made monthly.

### Survey Development

The survey was developed by an interdisciplinary team of experts in digital mental health, psychiatry, and clinical psychology. We did not use validated psychometric instruments or a specific theoretical framework. Item development was instead informed by a literature review of the current landscape of AI in mental health. The items went through multiple rounds of review by the research team to ensure clarity and relevance. AI was intentionally left undefined to capture broad subjective interpretations of the technology as it currently exists in public discourse. The finalized 12-item instrument covered 6 domains: clinical background, AI familiarity and adoption intent, perceived applications, barriers and ethics, training and equity, and open-ended feedback. Response formats included multiple choice, Likert-type scales, check-all-that-apply items, and 1 open-ended item. The survey was internally pretested for clarity, usability, and face validity. The 6 domains covered by the finalized instrument were as follows:

- Clinical background—highest academic or professional credential
- AI familiarity and adoption intent—current use, anticipated integration timelines, and openness to adoption
- Perceived applications—ratings across domains such as intake, triage, decision support, documentation, supervision, chatbot tools, and biomarker monitoring
- Barriers and ethics—risks including cost, integration issues, lack of administrative support, bias, privacy, and job displacement
- Training and equity—interest in AI-specific training and views on AI’s impact on disparities
- Open-ended feedback—optional free-text reflections

### Data Collection

The survey was administered via Qualtrics (Qualtrics International Inc) [13] optimized for desktop and mobile, with options to pause and resume. Reminders were sent via recruitment channels. No identifying information was collected. Data were stored on encrypted, password-protected servers accessible only to the research team.

### Analysis

Quantitative data were analyzed using the built-in Stats iQ module within the Qualtrics platform. To maintain transparency and reproducibility, statistical handling was defined. Missing data were managed via pairwise deletion, ensuring maximum retention of available data for each specific analysis without discarding entire cases. Survey items formatted as “select all that apply” were independently coded as mutually exclusive binary variables (selected vs unselected) for analysis. Before inferential testing, statistical assumptions were evaluated. For all chi-square tests of independence, expected cell frequencies were verified to ensure that they exceeded 5; where assumptions were met, standard unranked tests were run. Chi-square tests of independence were used to explore associations between AI use and other variables such as comfort levels, endorsement of clinical applications, and views on AI’s impact on care disparities. We evaluated effect size using the Cramér *V* and set significance at a *P* value below .05. No α-level adjustments were made for multiple comparisons. Consequently, these inferential analyses were exploratory and hypothesis generating.

Responses to the open-ended question were exported as a CSV file, and a descriptive qualitative summary approach was taken. One researcher reviewed all exported open-ended responses and conducted initial coding to organize the text into preliminary topical categories. Following this initial step, a second researcher reviewed the raw responses alongside the proposed categories. The 2 researchers discussed the coding structure and reached a consensus to finalize 6 overarching thematic categories.

## Results

### Respondents

A total of 62 licensed or trainee MHPs who were actively engaged in clinical care participated in the survey (Table 1). Most (n=35, 56.5%) held a master’s degree, whereas 33.9% (n=21) were PhD-level MHPs. In total, 6.5% (n=4) of the respondents primarily worked in administrative or leadership positions in mental health care but still maintained clinical responsibilities. Additional respondents included trainees (n=2, 3.2%).

**Table 1:** Breakdown of survey respondents by highest professional credential (N=62).

Professional credential	Participants, n (%)
Licensed Master’s-level clinician	35 (56.5)
Licensed PhD-level clinician	21 (33.9)
Administrator	4 (6.5)
Trainee	2 (3.2)
MD or DO	0 (0)
Nurse practitioner	0 (0)

Regarding current use of AI, most respondents (43/62, 69.4%) reported not using any form of AI in their clinical practice. When asked about anticipated integration timelines, the largest proportion of respondents (25/61, 41%) indicated that they were undecided. A smaller subset (13/61, 21.3%) had already begun incorporating AI tools into their practice. Other respondents planned to adopt AI in the next 6 (5/61, 8.2%), 7 to 23 (1/61, 1.6%), 24 to 35 (1/61, 1.6%), and ≥36 (1/61, 1.6%) months. Notably, nearly one-quarter (15/61, 24.6%) stated that they did not intend to adopt AI at all.

### Perceived Applications and Comfort

Participants were asked to evaluate various domains in which AI could be applied within mental health care (Table 2). The most widely endorsed application was for administrative functions such as coding and billing, with over half (32/60, 53.3%) of the respondents supporting this use case. Additional perceived applications of AI included intake and triage, automated documentation and progress note generation, decision support for diagnosis and treatment, chatbot-based patient support tools, and monitoring of behavioral or biological markers (Table 3). In the “Other” option of this survey item, several participants also identified unique or emergent areas where they believed AI could be beneficial, including patient education, marketing and outreach, scheduling, virtual care, clinical training, and protocol monitoring.

**Table 2:** Frequencies and percentages of respondents’ perceptions of the use of AI in mental health care based on a survey of US mental health professionals conducted from August 2024 to November 2024 (n=61).

Domain and response option		Participants, n (%)
AI integration timeline within practice		
	I already am incorporating AI into my practice	13 (21.3)
	In the next 1-6 mo	5 (8.2)
	In the next 7-23 mo	1 (1.6)
	In the next 24-35 mo	1 (1.6)
	In the next ≥36 mo	1 (1.6)
	Undecided	25 (41)
	Never	15 (24.6)
Areas of concern regarding the use of AI in mental health care (multiple selections possible)		
	Inaccurate outcomes or hallucinations	47 (77)
	Hidden bias	41 (67.2)
	Accessibility concerns for certain populations	31 (50.8)
	Personal job stability	22 (36.1)
	Other	19 (31.1)
Barriers to the adoption or implementation of AI in clinical care over the next 24 mo (multiple selections possible)		
	Clinician behavior or resistance to change	46 (75.4)
	Unclear clinical validation or efficacy	41 (67.2)
	Technical integration with existing systems	26 (42.6)
	Financial	25 (41)
	Payer reimbursement	24 (39.3)
	Purchasing complexity or confusion	24 (39.3)
	Support from administration	16 (26.2)
	Other	8 (13.1)
Comfort level using AI tools to assist in assessments and treatment planning		
	Very comfortable	6 (9.8)
	Somewhat comfortable	11 (18)
	Neutral	8 (13.1)
	Somewhat uncomfortable	19 (31.1)
	Very uncomfortable	17 (27.9)
Potential AI benefits in clinical mental health care		
	Reduction in clinical workload	19 (31.1)
	Increased efficiency in diagnosis and treatment	10 (16.4)
	Enhanced data analysis and patient insights	9 (14.8)
	Improved accessibility to care	5 (8.2)
	Other	18 (29.5)
Importance of clinicians receiving training on the ethical and practical use of AI in mental health care		
	Extremely important	26 (42.6)
	Very important	20 (32.8)
	Moderately important	8 (13.1)
	Slightly important	3 (4.9)
	Not at all important	4 (6.6)
Extent to which AI can help reduce disparities in access to mental health care across different populations		
	To a great extent	6 (9.8)
	To a moderate extent	15 (24.6)
	To a small extent	13 (21.3)
	Not at all	11 (18)
	Unsure	16 (26.2)

**Table 3:** Frequencies and percentages of respondents identifying specific clinical domains as promising areas for AI implementation within the next 24 months based on a cross-sectional survey of US mental health professionals conducted from August 2024 to November 2024.

Area		Yes, n (%)	No, n (%)	Maybe, n (%)
Administrative				
	Coding or billing (n=60)	32 (53.3)	7 (11.7)	21 (35)
	Automated intake (n=61)	23 (37.7)	21 (34.4)	17 (27.9)
	Self-help for low-acuity cases (n=61)	20 (32.8)	21 (34.4)	20 (32.8)
	Planning (n=61)	20 (32.8)	19 (31.1)	22 (36.1)
	Screening or triage (n=61)	18 (29.5)	27 (44.3)	16 (26.2)
Ambient listening				
	Documentation (n=61)	23 (37.7)	24 (39.3)	14 (23)
	Clinician “nudges” (n=61)	16 (26.2)	30 (49.2)	15 (24.6)
	Clinician training and supervision (n=60)	11 (18.3)	31 (51.7)	20 (33.3)
Clinical decision support (n=61)				
	Diagnosis	17 (27.9)	20 (32.8)	24 (39.3)
	Treatment	16 (26.2)	23 (37.7)	22 (36.1)
	Monitoring biomarkers	19 (31.1)	21 (34.4)	21 (34.4)
	Messaging with clients	17 (27.9)	26 (42.6)	18 (29.5)
Chatbots (n=61)				
	With a licensed clinician involved	14 (23)	27 (44.3)	20 (32.8)
	Without a licensed clinician involved	7 (11.5)	48 (78.7)	6 (9.8)

A chi-square test revealed that whether MHPs used AI in clinical practice significantly influenced their comfort using AI tools for assessment and treatment planning (n=61, *χ*^2^_4_=10.2; *P*=.04; Cramér *V*=0.401). Among those using AI, 55.6% (10/18) reported feeling either somewhat or very comfortable with such tools compared to only 16.3% (7/43) of nonusers. Conversely, discomfort was more common among nonusers, with 67.4% (29/43) of participants reporting being either somewhat or very uncomfortable using AI. This suggests that AI users are more likely to express confidence in integrating AI into clinical workflows (Table 4).

**Table 4:** Chi-square tests of association between current clinical use of AI and agreement with specific applications in mental health care based on a cross-sectional survey of US mental health professionals (MHPs) conducted from August 2024 to November 2024 (n=61).

Area			Chi-square (*df*)		*P* value		Cramér *V*
Administrative							
	Coding or billing	6.0 (2)		.049		0.317	
	Automated intake	13.0 (2)		.002		0.461	
	Self-help for low-acuity cases	4.7 (2)		.10		0.276	
	Planning	18.8 (2)		<.001		0.556	
	Screening or triage	13.2 (2)		<.001		0.465	
Ambient listening							
	Documentation	17.6 (2)		<.001		0.537	
	Clinician “nudges”	7.8 (2)		.02		0.358	
Clinician decision support							
	Diagnosis	14.7 (2)		<.001		0.490	
	Treatment	21.6 (2)		<.001		0.595	
	Monitoring biomarkers	11.7 (2)		.003		0.438	
	Messaging with clients	10.0 (2)		.007		0.404	
	Clinician training and supervision	8.3 (2)		.02		0.372	
Chatbots							
	With licensed MHPs	6.9 (2)		.03		0.336	
	Without licensed MHPs	14.5 (2)		<.001		0.488	

### Concerns and Ethical Considerations

When asked about respondents’ concerns about the use of AI in clinical practice, the most frequently cited were inaccurate outcomes (47/61, 77%) and algorithmic bias (41/61, 67.2%), followed by reduced accessibility for marginalized populations (31/61, 50.8%) and threats to job stability (22/61, 36.1%). Beyond these predefined options, respondents cited additional concerns in the “Other” option. MHPs voiced skepticism about the overuse of chatbots for therapeutic engagement, warning that excessive reliance on such tools could lead to the dehumanization of care. Several respondents highlighted the human connection in the therapeutic process and cautioned against treating AI as a substitute for relational work. Specific concerns included threats to patient privacy, regulatory breaches, and loss of emotional nuance in communication. Further comments suggested that AI tools were perceived as lacking sophistication, emotional intelligence, and linguistic precision. Several respondents also expressed concern about AI’s inability to comprehend complex human experiences, particularly in contexts involving trauma or relational dynamics. Ethical ambiguity surrounding the division of responsibility between MHPs and machines was another theme, as was the potential for cybersecurity breaches in sensitive clinical data environments. One seasoned MHP, reflecting on nearly 5 decades of experience, mentioned that the use of AI contradicts the foundational principles of therapeutic care. A large majority (54/61, 88.52%) rated formal training on the ethical and practical use of AI as important, underscoring the need for competency development before integration.

In response to whether AI could reduce disparities in access to mental health services, responses were mixed. While many participants acknowledged the potential for AI to improve access, some were less enthusiastic (Table 2). Overall, most MHPs felt that AI had the potential to reduce disparities to a “moderate” or “great” extent, although this optimism was tempered by recognition of the need for intentional, equitable implementation strategies. A chi-square test revealed a significant association between current use of AI in clinical practice and belief that AI improves access to mental health care across populations (n=61, *χ*^2^_4_=14.0; *P*=.007; Cramér *V*=0.479). Among current users of AI, 61.1% (11/18) believed that AI improves access either moderately or to a great extent, whereas only 23.3% (10/43) of nonusers shared that view. Conversely, most nonusers felt that AI had no benefit, were unsure, or felt that it had a small benefit. These findings suggest that MHPs who engaged with AI were more likely to view it as a tool for improving equity in care delivery.

### Barriers to Adoption

MHPs were asked what they felt were the most significant barriers to the adoption of AI in clinical care over the following 2 years. The most frequently selected challenge was MHP resistance to change or reluctance to modify established workflows (46/61, 75.4%). A closely related concern was uncertainty regarding the clinical validation and efficacy of AI tools (41/61, 67.2%), highlighting a hesitancy to integrate AI into practice without robust, peer-reviewed evidence of benefit. Technical integration challenges also featured prominently, with embedding AI systems into existing electronic health records and digital infrastructure being the most cited challenge among participants (26/61, 42.6%). Financial concerns were also mentioned (25/61, 41%) surrounding the cost of acquiring, maintaining, and updating AI systems in budget-constrained environments. Complexity or confusion surrounding the purchasing process was also mentioned (24/61, 39.3%), as was ambiguity regarding payer reimbursement structures for AI-supported care (24/61, 39.3%). Organizational readiness emerged as another constraint. Just over one-quarter of respondents (16/61, 26.2%) indicated that a lack of administrative support or leadership buy-in could pose a barrier to AI implementation.

In the “Other” option, several responses underscored the role of institutional culture in either facilitating or impeding innovation. Beyond structural and financial concerns, MHPs also expressed apprehension about patient acceptance. Some respondents noted that clients may be concerned about data privacy, cybersecurity, or surveillance when AI is used in clinical contexts. Others highlighted concerns about liability for incorrect AI-generated diagnoses or treatment recommendations. These concerns reflect a broader mistrust not only of the technology itself but also of the broader ecosystem into which it would be introduced. Respondents also pointed to the need for clearer delineation of clinical responsibility when AI tools are involved in decision-making. Several MHPs emphasized the importance of preserving therapeutic integrity, cautioning that overly rapid implementation without ethical safeguards, end user training, and patient education could risk doing more harm than good.

### Open-Ended Responses

#### Overview

The final item of the survey was an open-ended question asking whether participants had any additional insights regarding their perceptions of the use of AI in clinical mental health care. This optional item yielded responses from 48.4% (30/62) of the participants. Descriptive qualitative summary resulted in six categories of responses: (1) ethics and privacy, (2) therapeutic integrity, (3) cultural and algorithmic bias, (4) philosophical and systemic critiques, (5) risk and accountability, and (6) implementation readiness. The open-ended responses varied in depth and richness, ranging from brief statements to detailed paragraphs. Most were concise, offering single-sentence feedback on specific concerns. However, several participants provided extensive commentary exploring the ethical and philosophical implications of AI in mental health care. These detailed responses highlighted complex issues regarding patient privacy, the therapeutic alliance, and risks to vulnerable populations. While apprehension was the primary focus, a few responses briefly acknowledged potential utility, suggesting that AI might help by increasing accessibility to information.

#### Ethics and Privacy

Respondents voiced concerns over data breaches, inadequate consent, and misuse of clinical data by third parties. One warned the following:

There is no true guarantee of privacy.... Someone with the money will buy a company that has the software and use it in a deeply violating way.

Several respondents questioned whether AI could be implemented ethically among vulnerable populations, calling such use “highly unethical” without safeguards.

#### Therapeutic Integrity

Many respondents emphasized AI’s lack of emotional intelligence, empathy, and nonverbal attunement, warning of depersonalization. One stated the following:

There is no replacement for human connection.... AI promotes the opposite of individualized treatment.

Respondents stressed that relational authenticity cannot be replicated by machines.

#### Cultural Competence and Bias

Several respondents noted AI’s inability to capture cultural nuance, risking biased recommendations and overlooking sociocultural context. In mental health, where cultural, spiritual, and community values shape care, such blind spots were viewed as potentially harmful, with risks of racialized or gendered misinterpretation.

#### Philosophical and Systemic Critiques

Beyond clinical and ethical concerns, several participants offered broader critiques of AI’s role in the health care system. They worried that AI tools could be used to prioritize efficiency and cost cutting over patient-centered care. One MHP wrote the following:

I am very concerned that AI will be used in a profit-oriented healthcare industry for efficiency at the cost of quality care.

#### Risk and Accountability

Several responses focused on legal and ethical responsibility in the event of AI-generated harm. MHPs raised critical questions about who would be held accountable for errors made by or under the guidance of AI systems, particularly in scenarios involving misdiagnosis, inappropriate treatment, or data misuse. These concerns reflect a broader uncertainty about liability frameworks.

#### Implementation and Literacy

Some respondents highlighted the practical challenges of AI implementation. Several noted that many MHPs remain hesitant or lack sufficient training to feel confident using AI. Others expressed a need for more concrete use cases or demonstrations to better understand how AI could be meaningfully and ethically applied in practice. The importance of clinician training, interdisciplinary dialogue, and transparent rollout strategies was emphasized as a prerequisite for acceptance.

#### General Insights

These findings provide critical insights into the differing perspectives of MHPs regarding AI integration. While a minority of the respondents acknowledged the potential of AI to enhance access and reduce disparities, particularly through tools that augment rather than replace clinician input, a general tone across responses was one of caution. Concerns about ethical integrity, loss of therapeutic depth, systemic misuse, and unclear responsibility suggest that successful AI implementation will depend not only on technological capacity but also on cultural sensitivity, professional oversight, and explicit ethical design.

## Discussion

### Principal Findings

#### Overview

This study evaluated MHP perceptions of AI in clinical practice among a small, self-selected convenience sample of licensed or trainee MHPs in the United States, focusing specifically on perceived benefits, risks, implementation barriers, and training needs. The findings revealed that, while clinicians see the utility of AI for administrative and logistical optimization, they maintain ethical and practical reservations regarding clinical deployment. These results demonstrate that therapeutic readiness lags behind technical capability, offering a road map for future AI design, implementation, and governance frameworks tailored specifically to mental health care.

#### Balancing Optimism With Caution

Some MHPs noted AI’s potential to improve administrative efficiency, reduce documentation burden, and expand access, particularly for lower-acuity populations, but optimism was tempered by discomfort and uncertainty. Most respondents were undecided or unwilling to adopt AI, mirroring broader digital health trends where technological potential outpaces provider readiness, especially in relational care contexts [12,14,15]. Consistent with prior research, MHPs saw AI as an adjunct, not a replacement, favoring logistical over interpretive or relational functions. The message was clear: mental health care is grounded in human connection and individualized understanding, which AI cannot replicate. This focus highlights a distinct divide between mental health and other medical specialties. In other fields, clinician acceptance of AI is seen more positively and is related more to objective technical accuracy, diagnostic precision, and image processing speed [16]. For MHPs, however, the primary benchmark is not algorithmic performance but preserving the therapeutic alliance. Because mental health care relies on subjective narrative interpretation, empathy, and relational safety rather than objective biomarkers, MHPs exhibit a unique skepticism centered on the potential dehumanization of care. While general medical clinicians may embrace AI for its interpretive capabilities, MHPs strictly relegate the technology to a supportive, administrative role.

#### Ethical, Cultural, and Relational Concerns

Concerns about ethics, privacy, consent, bias, and misuse of sensitive data were prominent, echoing wider AI ethics debates [17-19]. The therapeutic alliance, central to mental health care, was seen as vulnerable to disruption, with doubts about AI’s capacity for empathy, nonverbal interpretation, or dynamic response to distress. MHPs warned that poorly designed AI could perpetuate systemic biases, particularly if trained on nondiverse datasets, reinforcing rather than reducing disparities [17-19]. While some saw potential for improving access, most emphasized the need for oversight to avoid exclusion.

#### Barriers to Adoption and the Role of Support

Key barriers included MHP resistance, lack of clinical validation, and poor integration with existing systems, challenges common in psychiatry and psychotherapy [2,15]. While skeptical, most participants supported specialized training on ethical and practical AI use, signaling openness if paired with adequate education and support. However, training alone is insufficient; implementation must also ensure workflow compatibility, usability, and institutional backing to achieve adoption.

#### Trust, Autonomy, and Clinical Judgment

Trust in AI and health systems was a recurring theme. Concerns about accountability, transparency, and liability for AI-generated errors highlight the need for governance frameworks. Several respondents advocated for patient choice in AI-mediated care, aligning with shared decision-making principles [20-22]. MHPs favored AI as a supportive tool, enhancing, not replacing, professional expertise.

### Implications for Practice and Policy

Effective AI integration will require relational sensitivity, ethical rigor, and cultural humility [23]. Co-design with MHPs, diverse validation studies, and transparent accountability structures are critical. MHP leadership is essential to safeguard therapeutic integrity, equity, and trust in digitally augmented care.

### Limitations and Future Directions

Limitations include a relatively small, self-selected sample and cross-sectional design, limiting generalizability across clinical populations and settings and introducing the risk of selection bias [24]. The quantitative analyses were exploratory in nature. Multiple comparisons were conducted without α-level correction, which increases the risk of type I errors. The inclusion of a single open-ended item, while allowing for some exploration of how and why respondents held certain views, limited the depth of insight that could be obtained. Future studies should use richer qualitative methods such as interviews or focus groups to explore these perspectives in greater depth. Furthermore, AI was intentionally left undefined in the survey to capture broad subjective interpretations of the technology as it currently exists in public discourse. While this choice allowed for a broader assessment of baseline attitudes, variations in how participants conceptualized AI constitute a limitation, as differing interpretations may have influenced specific ratings and comfort levels. Another limitation is that the survey was not formally pretested with a representative sample following subsequent post hoc revisions. Only the research team reviewed these later changes.

Future research should track how attitudes shift with exposure, training, and real-world use and examine impacts on clinical outcomes, professional identity, and patient engagement. Studies should also prioritize perspectives from marginalized providers and communities to ensure equitable AI development and deployment.

### Conclusions

MHPs are neither wholly resistant nor uncritically accepting of AI in mental health care. Instead, they offer a balanced perspective that values administrative utility while fiercely protecting the relational depth of clinical practice. The integration of AI in psychiatry and psychotherapy is ultimately a human challenge rather than a technical one, deeply intertwined with professional identity and systemic equity. Because mental health care relies uniquely on the therapeutic alliance, implementation models from broader medicine cannot simply be copied and pasted into this field. Responsible innovation will require dedicated clinical co-design, transparent liability frameworks, and proactive training to ensure that digital tools support rather than compromise patient-centered care.

## Data Availability

Data are available upon request.

## Abbreviations

MHP: mental health professional
